# Post-eruptive mobility of lithium in volcanic rocks

**DOI:** 10.1038/s41467-018-05688-2

**Published:** 2018-08-13

**Authors:** B. S. Ellis, D. Szymanowski, T. Magna, J. Neukampf, R. Dohmen, O. Bachmann, P. Ulmer, M. Guillong

**Affiliations:** 1 Institute of Geochemistry and Petrology, ETH Zürich, NW Clausiusstrasse 25, 8092 Zürich, Switzerland; 2 Czech Geological Survey, Klárov 3, 11821 Prague 1, Czech Republic; 3 Institute of Geology, Mineralogy and Geophysics, Ruhr-University Bochum, Bochum, 44780 Nordrhein-Westfalen Germany

## Abstract

To reflect magmatic conditions, volcanic rocks must retain their compositions through eruption and post-eruptive cooling. Mostly, this is the case. However, welded ignimbrites from the Yellowstone–Snake River Plain magmatic province reveal systematic modification of the lithium (Li) inventory by post-eruptive processes. Here we show that phenocrysts from slowly cooled microcrystalline ignimbrite interiors consistently have significantly more Li than their rapidly quenched, glassy, counterparts. The strong association with host lithology and the invariance of other trace elements indicate that Li remains mobile long after eruption and readily passes into phenocrysts via diffusion as groundmass crystallisation increases the Li contents of the last remaining melts. Li isotopic measurements reveal that this diffusion during cooling combined with efficient degassing on the surface may significantly affect the Li inventory and isotopic compositions of volcanic rocks. Utilisation of Li for petrogenetic studies is therefore crucially dependent on the ability to ‘see through’ such post-eruptive processes.

## Abstract (web summary)

Lithium, an increasingly economically important element, is also used to trace the cycling of materials through the Earth system. Here the authors show that post-eruptive processes such as degassing and groundmass crystallisation control the inventory of lithium in volcanic deposits.

## Introduction

Understanding global geochemical cycling of elements between different reservoirs is crucial to understanding how the Earth system works^1^. Constraining elemental cycling in turn requires the ability to trace various components (e.g., rocks, sediments, or fluids) as they are processed via plate tectonics^2^. In the past two decades, lithium (Li) isotopic systematics have been increasingly used to complement more traditional isotopic tracers (O, Sr, Pb, Nd, Hf) of petrogenesis^3–7^. The large mass difference of ~16% between the two isotopes (^6^Li, ^7^Li) renders them particularly susceptible to isotopic fractionation at low temperatures^8,9^ while magmatic differentiation processes are inferred not to cause significant Li isotopic fractionation^10,11^. Other studies have used Li abundances and isotopic compositions to estimate the input from sediments to subduction zones^5,12–15^. At shallower depths, the rapid diffusion of Li allows it to capture processes occurring in volcanic systems at the syn- and even post-eruptive stages^16–20^. Yet, the extent to which these post-eruption processes modify the original Li isotopic signature of the deposit remains poorly known.

**Fig. 1:**
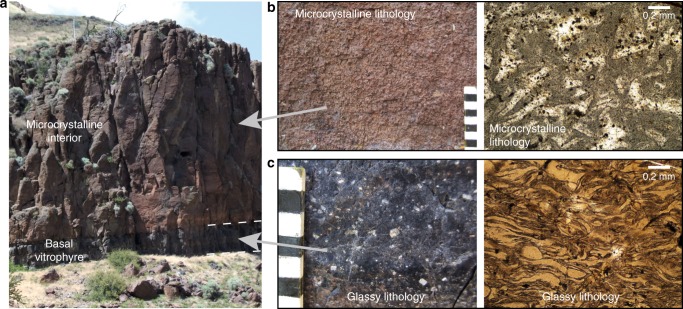
Typical variability of groundmass crystallinity in high-grade welded ignimbrites. Glassy portions of the deposits (**a**, **c**) are preserved at the rapidly cooled upper and basal contacts while the slowly cooled central portions of the deposit are characterised by microcrystalline groundmass (**a**, **b**). Divisions on ruler are 1 cm, outcrop height ~15 m

To address this, we analysed a suite of pristine, welded ignimbrites that are produced from individual pyroclasts agglutinating together following eruption and deposition, a process favoured by high pyroclast temperatures and rapid accumulation^21^. In the field, these welded ignimbrites share a common lithological stratigraphy with glassy vitrophyres at the base and top of the unit, where the hot mass of pyroclasts rapidly chilled against the ground or atmosphere, and a central microcrystalline portion in which cooling progressed more slowly^21^ (Fig. 1). Following high-temperature emplacement, many of these welded ignimbrites underwent rheomorphism (i.e., ductile deformation at the surface) preserving deposits with spectacular folds. The rheomorphism underscores that these deposits indeed were magmatic liquids on the surface for prolonged periods (i.e., up to tens of years^22^) following eruption.

**Fig. 2:**
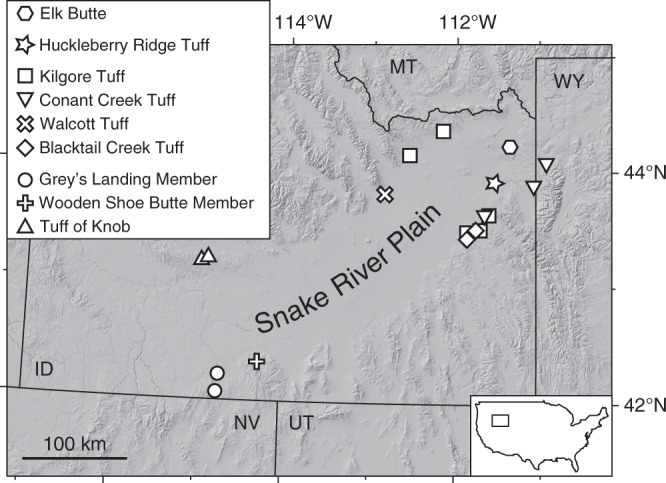
Digital elevation map of the Snake River Plain area and sampling locations of analysed rhyolites. ID Idaho, MT Montana, WY Wyoming, NV Nevada, UT Utah. Base map is a processed NASA Shuttle Radar Topography Mission (SRTM) v4.1 90 m digital elevation model (DEM)^23^

The compositionally bimodal (basalt–rhyolite) Yellowstone–Snake River Plain (YSRP) system in north-western North America (Fig. 2) represents the youngest and best-preserved continental large igneous province. The rhyolitic ignimbrites from the YSRP are commonly intensely welded and well exposed on the northern and southern margins of the Snake River Plain (SRP). The magmas from the SRP and, to a lesser extent, Yellowstone that produced these deposits were hotter (>850 °C) and drier (<3 wt.% H_2_O) than typical arc rhyolites^24,25^, a feature reflected in their anhydrous mineralogy typically consisting of plagioclase ± sanidine ± quartz + pyroxenes ± Fe–Ti oxides, and accessory zircon and apatite. Individual crystals commonly show limited compositional variability in major elements^25–27^. The eight well-characterised ignimbrites studied here span the past ~10 Myr of silicic volcanism from the central Snake River Plain to the Yellowstone Plateau (Fig. 2) and encompass much of the compositional variability in the rhyolitic ignimbrites of the Yellowstone province. The Tuff of Knob, Wooden Shoe Butte Member and the Grey’s Landing Member are taken as representative of the central SRP^22,28^. From the 6.6–4.0 Ma Heise volcanic field, the four main ignimbrites, the tuffs of Blacktail Creek, Walcott, Conant Creek and Kilgore, have been investigated^29–31^, and from the Yellowstone volcanic field the ~2 Ma Huckleberry Ridge Tuff^32–34^ has been selected. Large-volume (>1000 km^3^) ignimbrites are represented by the Kilgore, Blacktail Creek and Huckleberry Ridge tuffs, while the Grey’s Landing and Tuff of Knob ignimbrites have volumes estimated at <100 km^3^ (see refs. ^29,35^).

Here we present new in situ Li and other trace element data from lithologically constrained samples to investigate the mobility of lithium in the post-depositional realm. These analyses are complemented by a detailed isotopic investigation of a typical ignimbrite which reveals that the post-eruptive mobility of lithium also has significant implications for the Li isotopic record preserved in volcanic deposits.

## Results

### Lithium abundances in phenocrysts

**Fig. 3:**
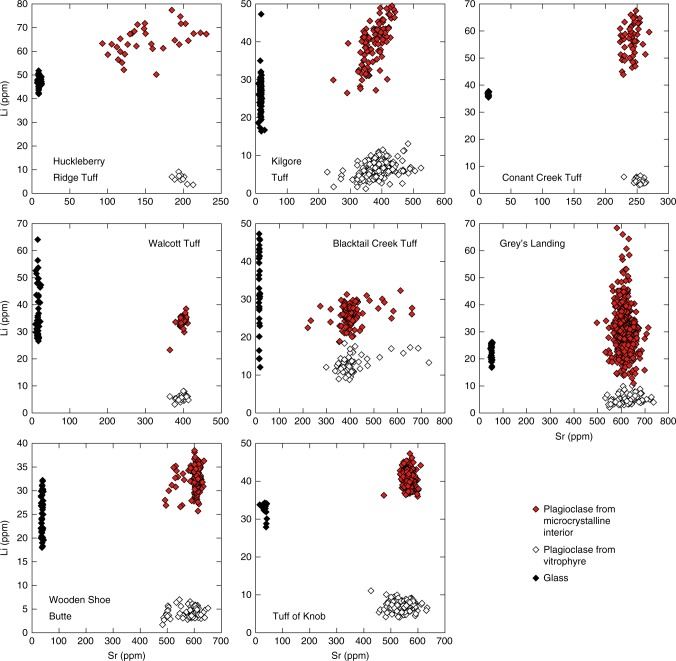
Lithium abundances in plagioclase crystals and glass from rhyolites considered in this study. Plagioclase crystals from the microcrystalline portion of the deposit have consistently higher Li contents than those from the glassy portions of the same deposit (full data in Supplementary Data 1). Typical uncertainties for Li and Sr are <5% (2σ). Grey’s Landing data are from ref. ^22^

In all of the eight ignimbrites considered here, a remarkably common pattern of Li distribution is observed. Feldspar phenocrysts from the rapidly cooled, glassy portions of the ignimbrite always have lower Li abundances than the equivalent crystals from the slowly cooled microcrystalline portion of the deposit (Fig. 3).

The increase in average Li content of slowly cooled relative to quickly cooled plagioclase crystals varies between ignimbrites from as little as a factor of two (Blacktail Creek Tuff) to approaching an order of magnitude (Walcott and Huckleberry Ridge tuffs; Fig. 3). The increase in Li content of plagioclase from microcrystalline lithologies is consistent across the eruptive units containing plagioclase of anorthite >40 (e.g., Tuff of Knob, Wooden Shoe Butte Member) as well as those with a range of feldspar compositions extending into the anorthoclase field (Kilgore Tuff). An intriguing feature of the plagioclase data is that the variability observed in Li is absent in other trace elements which, while showing variability between ignimbrites, are identical between different portions of the same deposit. Interestingly, a similar behaviour is also observed in other mineral phases within the rhyolitic ignimbrites. Sanidine, augite and pigeonite phenocrysts all exhibit systematically lower Li contents in glassy lithologies than in their respective microcrystalline equivalents with other trace elements unaffected (illustrated in Supplementary Fig. 1, 2). In contrast to the other phases investigated, quartz crystals from the Huckleberry Ridge and Blacktail Creek tuffs contain identical Li contents regardless of the post-eruptive cooling regime (Supplementary Fig. 3).

### Li isotopic compositions

**Fig. 4:**
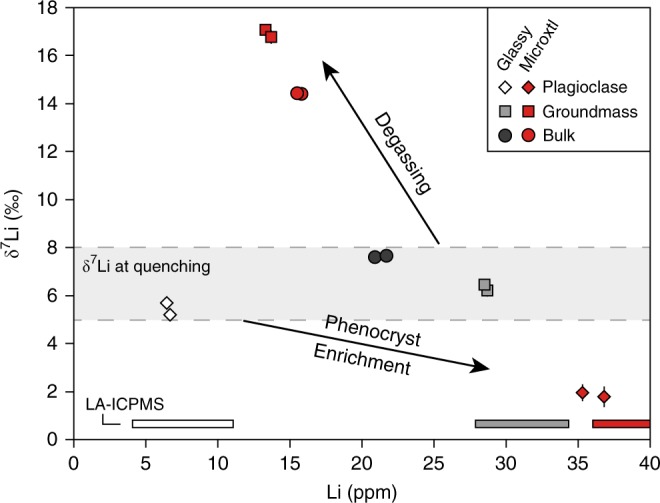
Lithium isotopic diversity in the Tuff of Knob. δ^7^Li values and Li abundances in bulk, groundmass and plagioclase from the Tuff of Knob vary as a function of cooling rate. Coloured bars along the abscissa indicate the total ranges of Li measured in situ in the respective phases

Lithium abundances and isotopic compositions were determined for the Tuff of Knob, which shows a particularly clear distinction in Li abundances (Fig. 3). For both quickly and slowly cooled lithologies, three sample types were analysed: (1) bulk ignimbrite, (2) plagioclase separates and (3) groundmass separates. For plagioclase and glass, concentrations determined by bulk mineral separate solution and in situ laser ablation inductively coupled plasma mass spectrometry (LA-ICPMS) are in excellent agreement (Table 1 and Fig. 4). The five samples from the vitrophyre (bulk, glass separates and feldspar separates) span a relatively restricted range in δ^7^Li values between 5.2‰ for plagioclase and 7.7‰ for bulk sample (Table 1), which is in accord with the limited Li isotopic fractionation suggested for high-temperature systems^5,10,36,37^.

**Table 1:** Lithium abundance and isotopic composition of bulk rock, groundmass and plagioclase from the Tuff of Knob

Sample	Li (ppm)	δ^7^Li (‰)	±2σ	LA-ICPMS Li range (ppm)
Microcrystalline interior				
Bulk	15.8	14.41	0.20	
Bulk repeat	15.5	14.44	0.19	
Groundmass 1	13.3	17.08	0.05	
Groundmass 2	13.7	16.78	0.28	
Plagioclase 1	36.8	1.79	0.43	36.0–47.3
Plagioclase 2	35.3	1.96	0.35	36.0–47.3
Vitrophyre				
Bulk	20.9	7.61	0.23	
Bulk repeat	21.7	7.67	0.24	
Glass 1	28.7	6.22	0.25	27.9–34.3
Glass 2	28.5	6.46	0.15	27.9–34.3
Plagioclase 1	6.7	5.21	0.14	4.1–11.1
Plagioclase 2	6.5	5.70	0.23	4.1–11.1

In stark contrast to the vitrophyric samples, the equivalent samples from the microcrystalline lithology encompass a δ^7^Li range of >15‰ (Fig. 4). Both the groundmass and the bulk sample have a lower Li abundance and higher δ^7^Li than samples from the glassy lithology (Table 1). Plagioclase from the microcrystalline portion of the deposit has much higher Li abundance and is isotopically significantly lighter (35.3–36.8 ppm Li, δ^7^Li = 1.8–2.0‰) than plagioclase from the vitrophyre (6.5–6.7 ppm Li, δ^7^Li = 5.2–5.7‰). The apparent fractionation factor *α*_plagioclase–melt_ = 0.985 calculated for the microcrystalline portion differs significantly from that calculated for the vitrophyre (*α*_plagioclase–melt_ = 0.999). These observations hint at a process depleting the centre of the ignimbrite in Li (in particular ^6^Li) and illustrate the effects of prolonged cooling on redistribution of Li in the microcrystalline part of the deposit on a cooling-related timescale^37^.

## Discussion

Understanding the mechanisms responsible for the vast movement of Li in the investigated ignimbrites (and potentially other volcanic rocks emplaced both subaerially and subaqueously) requires a clear distinction between magmatic (i.e., pre-eruptive) processes and post-eruptive processes occurring during and after the emplacement. Our new dataset employing in situ Li analyses of both rapidly and slowly cooled portions of individual deposits combined with Li isotopic data from the Tuff of Knob allows us to clearly distinguish the influence of the two environments. In all studied units, there is no major elemental difference between bulk compositions of the rapidly cooled vitrophyres and the slowly cooled centres of ignimbrites, nor is there any contrast in major or trace element composition of feldspar between the two cooling domains (see Supplementary Data 1). The only exception to this is Li, which is considerably depleted in the bulk of the microcrystalline portion (see Fig. 4 for Tuff of Knob) but relatively enriched in the feldspars (Figs. 3, 4). When considered together, these points make a compelling case that the variability in Li in these eight ignimbrites cannot reflect a magmatic (i.e., pre-eruptive) process, but rather a subsequent process related to the difference in cooling histories of the glassy and microcrystalline lithologies.

In the Tuff of Knob, the Li abundance data may be considered together with isotopic data (Fig. 4). Based on the limited isotopic diversity in the glassy samples we consider these values to define the Li abundances and isotopic ratios immediately upon deposition (and to best approximate the original magmatic values), whereas the samples from the microcrystalline interior are considered to record post-emplacement processes (e.g., ref. ^30^). The decrease in Li abundance and increase in δ^7^Li in the bulk and groundmass microcrystalline samples (Fig. 4) may be interpreted to reflect the effects of post-emplacement degassing which causes an overall loss of Li to the atmosphere, and an increase in δ^7^Li dominantly through kinetic isotopic fractionation^38–40^. Diffusive fractionation of stable isotopes during bubble growth has recently been shown^41^ to exceed 5‰ for Cl with even greater effects predicted where the isotope pairs have larger differences in diffusivities as is the case for ^6^Li and ^7^Li (ref. ^42^). The central, microcrystalline parts of the deposit cool slowly and may stay hot for months to years after deposition^22^, allowing for efficient degassing, which is often recorded by vesicular portions of the ignimbrites^21^ (illustrated in Supplementary Fig. 4). Bulk measurements of the Tuff of Knob suggest that this degassing caused a loss of approximately 25% of the total Li inventory.

The phenocryst data, however, require a process separate from degassing as crystals from the slowly cooled interior of the Tuff of Knob exhibit a higher overall Li abundance (by a factor of ~5) in addition to an isotopically lighter Li signature compared to that from the vitrophyre (Figs. 3, 4). We re-iterate here that the major element compositions of feldspars within any single unit are identical between the different lithologies, allowing any potential control on Li partitioning into feldspar based on anorthite dependence^43,44^ to be discounted. Given the strong association between microcrystalline lithology and both Li abundance and low δ^7^Li in plagioclase, we consider that these signals must be generated via the process of post-emplacement crystallisation of a rhyolitic liquid at the surface, likely associated with expeditious diffusion of Li into plagioclase^16,36^.

**Fig. 5:**
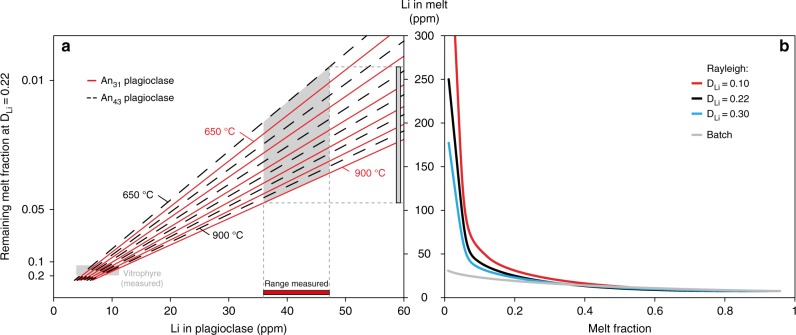
Models of conditions necessary for Li enrichment in plagioclase within the slowly cooled portions of the Tuff of Knob. **a** Equilibrium relationship between plagioclase and melt Li contents using the partitioning model of Dohmen and Blundy^44^. Shown here are calculations with the most anorthitic (An_43_) and average (An_31_) plagioclase compositions with temperatures ranging from 900 °C to 650 °C at 50 °C intervals. These calculations assume a constant D_Na_ of 2.52, based on the glassy Tuff of Knob sample. Data from ref. ^47^ indicate that even when groundmass crystallisation is almost to completion, the D_Na_ does not differ significantly (2.73 for Elk Butte). **b** Batch and Rayleigh fractionation models to predict the evolution of lithium content in the remaining melt as groundmass crystallisation progresses. Illustrated are a range of bulk partition coefficients for Li, but regardless of the value chosen, to reach the elevated values shown in **a** requires a Rayleigh fractionation process

Modelling of plagioclase-melt partition coefficients using the model of Dohmen and Blundy^44^ indicates that while the Li contents of crystals in the vitrophyre are broadly predictable from the Li content of the co-existing glass, the Li contents of the plagioclase crystals in the microcrystalline lithology cannot be generated from melts with Li contents similar to those preserved in the vitrophyre glass (Fig. 5). Partitioning models using the crystal compositions (average and maximum anorthite contents) and temperatures between 900 and 650 °C indicate that these crystals would be in equilibrium with melts of between 100 and 250 ppm Li (Fig. 5), significantly higher than the typical glass contents of ~50 ppm found in YSRP rhyolites (Fig. 3).

**Fig. 6:**
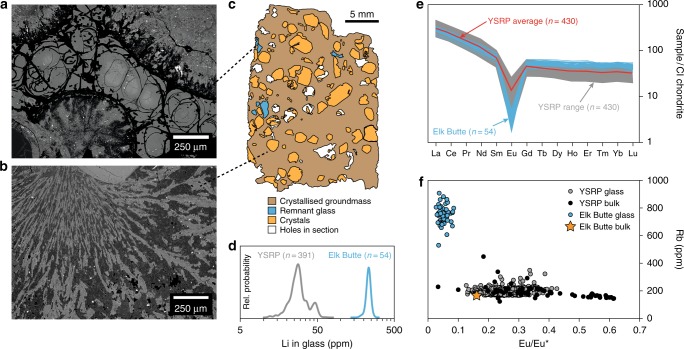
Composition of last remnant glass prior to complete groundmass crystallisation (Elk Butte dome of the Island Park series^47^). Small slivers of glass (**a**) represent the last remaining liquids in a dominantly crystallised groundmass (**b**). The remnant melt pockets in Elk Butte (**a**, **c**) have Li contents of the same magnitude as predicted in Fig. 5 (**d**) illustrating the geological reality of such a process. While these remnant glasses have similar characteristics to typical Yellowstone glasses (**e**), their trace elements reflect the extended crystallisation of the groundmass with elevated incompatible elements such as Li and Rb (**f**)

Our results (Fig. 3 and Supplementary Figs. 1–3) indicate that Li is incompatible to varying degrees in all of the phases crystallising in rhyolitic magmas. Therefore, with progressive crystallisation of the groundmass (dominated by sanidine, quartz and plagioclase^22^), the last remaining pockets of liquid should be enriched in Li. To test this we modelled the evolution of the Li content of melt in the Tuff of Knob using both batch and Rayleigh fractionation models (Fig. 5). The Rayleigh fractionation models suggest that with extensive crystallisation the Li content of the last remaining melt can be significantly increased. This is similar to extensive fractionation of granitic melts where the final pegmatitic liquids often produce Li-bearing mineral phases at sub-magmatic temperatures^45,46^. In the welded ignimbrites of the Snake River Plain, the contacts between the glassy vitrophyres and the microcrystalline interiors are typically sharp (Fig. 1) with the process of crystallisation going to completion in the interiors, resulting in poor preservation of these last remaining liquids. Fortuitously, within the Yellowstone system, the small-volume, Elk Butte dome^47^ contains small slivers of glass within a dominantly crystallised groundmass (Fig. 6). These ‘remnant glasses’ are similar in major elemental composition to fully glassy rhyolites from the YSRP province (average SiO_2_ 77.5 wt.%, Na_2_O 3.0 wt.% and K_2_O 5.1 wt.%)^47^. In terms of trace elements, they exhibit similar rare earth element (REE) patterns to typical YSRP rhyolites, but with a markedly more negative Eu anomaly (Fig. 6). Lithium contents of these glasses are between 188 and 270 ppm (average 236 ppm, *n* = 54), which is similar to the values required by the partitioning calculations for high-Li plagioclase in the Tuff of Knob (Fig. 5). Rubidium (Rb) contents are also notably elevated (average 756 ppm), while Sr contents are lower than typical YSRP rhyolites, leaving Rb/Sr ratios an order of magnitude greater than from entirely glassy samples. Despite the fourfold increase in Rb above the levels typical of YSRP glasses, in contrast to the behaviour of Li, plagioclase crystals from the slowly cooled interiors of ignimbrites are not elevated in Rb when compared to their rapidly cooled counterparts. The similarity of the measured Li contents in the ‘remnant glasses’ from Elk Butte provides additional strong support for the role of Rayleigh fractionation during post-eruption crystallisation of the Tuff of Knob (Fig. 5).

While the increase in the abundance of Li within the feldspars may be explained by the extended fractionation of the groundmass, their significant isotopic disequilibrium (δ^7^Li~14‰, Fig. 4) when compared to groundmass samples requires further consideration. Assuming limited change in the equilibrium isotopic fractionation between the rapidly cooled and slowly cooled plagioclase populations and their respective melts, the isotopic shift observed in feldspars from the microcrystalline core (δ^7^Li = 1.9 ‰ vs. 5.2–5.7‰ in the vitrophyre) must result dominantly from kinetic fractionation as Li diffuses into plagioclase with faster ^6^Li diffusion^16,48–50^. Based on calculations in Tomascak et al.^51^ using diffusion coefficients given in Giletti and Shanahan^52^, plagioclase crystals of 100 µm diameter would be expected to have fully re-equilibrated within days or less. That such equilibrium is not achieved hints that the experimentally determined diffusion coefficients^52^ for Li in plagioclase may be in error. We speculate that this may be the result of these experiments using isotopic tracer diffusion rather than chemical diffusion, which may be limited by the coupling of Li to slower diffusing elements. Large variations in Li abundances (up to an order of magnitude within single plagioclase crystals) have been reported from other slowly cooled deposits^22,40,53^, which are equally difficult to reconcile with rapid diffusional re-equilibration of Li in plagioclase. Given the uncertainties associated with the cooling rate of the deposit, the role played by concurrent degassing and the range of crystal sizes, we do not attempt to further constrain Li diffusion rates in plagioclase here. However, our data may provide motivation to address this question rigorously through experimental petrology.

The recognition that Li isotopic ratios may be modified well into the post-eruptive cooling history of a volcanic deposit calls for additional care to be taken when interpreting Li isotopic results. For volcanic processes, it is widely assumed that quenching of juvenile clasts on fragmentation effectively ‘locks in’ the signal preserved in volatile elements such as Li^20,22,54^. Yet, even this assumption may not be entirely robust as melt inclusions from centimetre-sized bombs from Volcán de Fuego, Guatemala, have been shown to lose H during post-fragmentation cooling^55^. A similar effect has been noted to affect mantle xenoliths, where xenoliths in andesitic pyroclastic deposits exhibited limited Li isotopic disequilibrium, while those entrained in more slowly cooled basaltic lavas showed a much wider isotopic variability^37^. Given that the products of all effusive and many explosive eruptions are not immediately quenched, the post-eruptive effects require careful consideration. Our findings suggest that the processes described in this study may act irrespective of the bulk composition of the system and that Li concentrations and isotopic ratios in bulk rock and minerals may not always faithfully record the Li inventory of a magma.

**Fig. 7:**
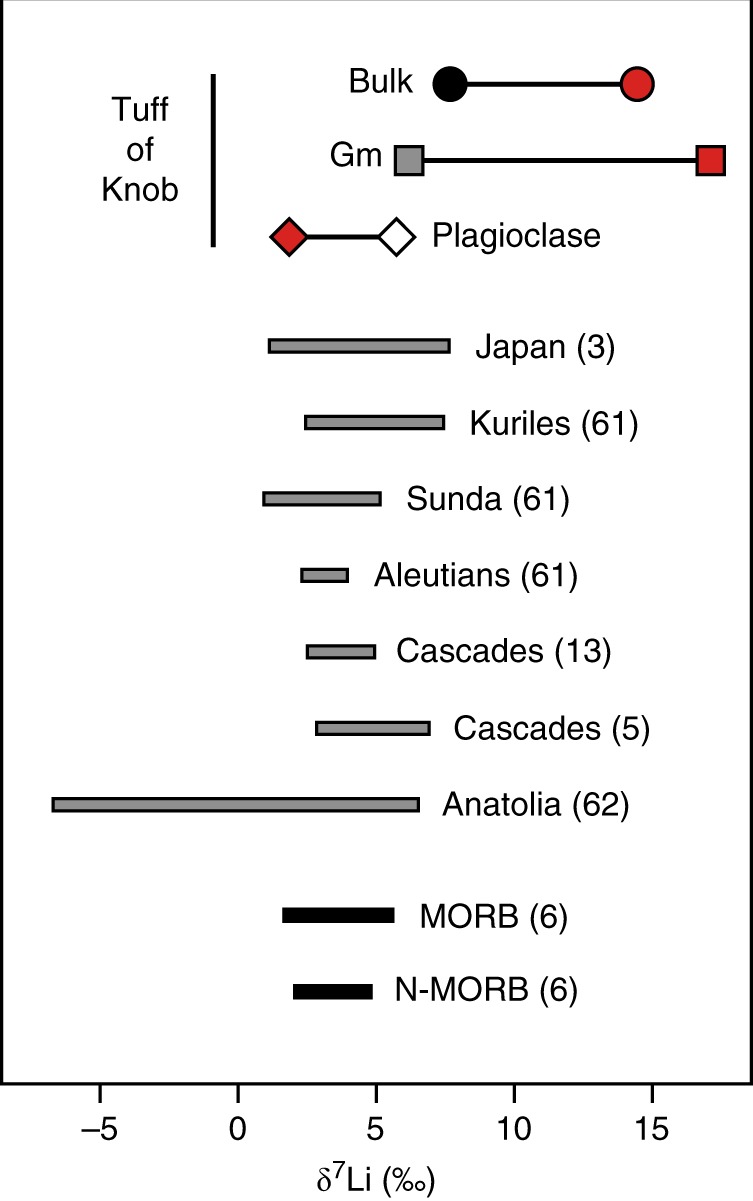
Variability in δ^7^Li in the Tuff of Knob compared to previous regional studies. Sources of Li isotopic data are marked with respective reference numbers

Bulk Li isotopic studies have investigated the history of material subducted back into the deeper mantle which affects Li isotopic signatures observed at mid-ocean ridges^4,6,8^, or attempted to fingerprint subducted materials in volcanic arcs^3,5,12,14^. Such approaches require that early, deep processes reflective of source regions are not overprinted by later, shallow (or indeed surficial) processes. The range of bulk δ^7^Li values from a single ignimbrite, here represented by the Tuff of Knob, is >6.5‰. Such Li isotopic variability exceeds that observed in many across-arc studies, which have interpreted the data as indicating variable proportions of slab-derived fluid and mantle wedge material^3,5,12^ (Fig. 7).

The time-consuming and challenging nature of Li isotopic analyses typically limits the number of analyses in a single study. Thus, most studies have preferred a wider geographic spread compared to more in-depth examinations of single deposits. By comparing samples from eight different rhyolitic ignimbrites in light of their post-emplacement cooling rates, we can make a compelling case for the post-eruptive modification of both Li abundances and isotopic compositions. These post-eruptive processes may generate Li isotopic heterogeneity which, without due regard for sample lithology, may partly or completely obscure a deeper (i.e. mantle) signature.

## Methods

### Trace elements in phenocrysts

Trace element concentrations in feldspars and glasses were determined in situ by LA-ICPMS using a 193 nm Resonetics ArF excimer laser coupled to a Thermo Element XR ICP mass spectrometer, housed at the Institute of Geochemistry and Petrology, ETH Zürich. Spot sizes varied with analytical session but typically 43 μm spots were used. The output energy of the laser beam was typically ∼3.5 J cm^−2^. The MATLAB-based program SILLS^56^ was employed to calculate the trace element concentration ratios using signal intensities obtained from NIST612 silicate glass external standards measured twice every 20–25 spots to correct for instrumental drift. For each data point the resulting elemental ratios were converted to absolute concentrations by using SiO_2_ as an internal standard. Previous work on all of the ignimbrites in this study allows the raw laser data from feldspar and glass analyses to be reduced using the appropriate silica content of the phase from the respective unit^31,57^. The USGS (United States Geological Survey) reference glass GSD-1G was used as a secondary standard to monitor the accuracy of the instrument. The precision of a single spot analysis is difficult to quantify, but replicate analyses of a homogeneous mineral or glass give precisions better than 5% of the value where element concentrations are significantly above the limit of detection.

Trace element concentrations in quartz were determined using a 193 nm GeoLas ArF excimer laser coupled to an Elan 6100 DRC quadrupole mass spectrometer, housed at the Institute of Geochemistry and Petrology, ETH Zürich. The output energy of the laser beam was 15–20 J cm^−2^. All quartz analyses were performed with a spot size of 60 μm. NIST 610 silicate glass was used as an external standard and a natural quartz crystal (ref. ^58^) was used as a secondary standard.

### Lithium isotopic analysis

Sample preparation and mass spectrometry analyses for Li isotopic compositions were performed at the Czech Geological Survey, Prague. For isotopic analyses of bulk rock, groundmass and plagioclase separates, samples were weighed into Savillex beakers and dissolved in a mixture of concentrated HNO_3_ and HF (1:6 v/v) at 130 °C for 72 h. Following decomposition, the sample solutions were dried, refluxed repeatedly with small amounts of concentrated HNO_3_ and equilibrated in 6 M HCl at 80 °C for 24 h. The analytical methods for Li isolation, purification and isotopic measurements of bulk rocks and mineral separates are detailed in ref. ^59^. The elution of Li from the matrix was accomplished using 80% methanol–1 M HNO_3_ mixture and BioRad AG50W–X8 (mesh 200–400) resin. Lithium concentrations and isotopic compositions were determined using a Neptune multiple-collector ICPMS (Thermo Scientific, Bremen, Germany). Lithium concentrations were measured in clean Li fractions against the L-SVEC reference solution^60^. A standard–sample–standard bracketing method using L-SVEC solution was employed to determine natural Li isotopic variations in unknown samples. The results of Li isotopic measurements are reported in the δ-notation relative to the L-SVEC reference solution and calculated as δ^7^Li (‰) = [(^7^Li/^6^Li)_sample_/(^7^Li/^6^Li)_L–SVEC_ −1] × 1000. The reproducibility was generally better than ±0.5‰ (2 SD), assessed from three to five individual runs. The reliability of analytical procedures was assessed using basalt BHVO-2, rhyolite JR-2 and basalt JB-2. The resulting δ^7^Li values for these reference materials were 4.42 ± 0.16‰, 4.04 ± 0.25‰ and 4.74 ± 0.62‰, respectively. These values are consistent with published data^4,5,10,37,50,59^. Replicate analysis of BHVO-2 yielded a δ^7^Li value of 4.66 ± 0.15‰, attesting to the reliability of the analytical methods.

### Data availability

All data generated during this study are included in this published article and its supplementary information files.

## Electronic supplementary material

Supplementary Information

Description of Additional Supplementary Files

Supplementary Data 1
