# Supplementary material for: Post-eruptive mobility of lithium in volcanic rocks
Source: Nat Commun. 2018 Aug 13;9:3228. doi: 10.1038/s41467-018-05688-2 (PMC6089988; doi:10.1038/s41467-018-05688-2)
Supplement: Supplementary file 1 — Supplementary Information [file 41467_2018_5688_MOESM1_ESM.pdf]

Supplementary Information

Post-eruptive mobility of lithium in volcanic rocks

B.S. Ellis et al.

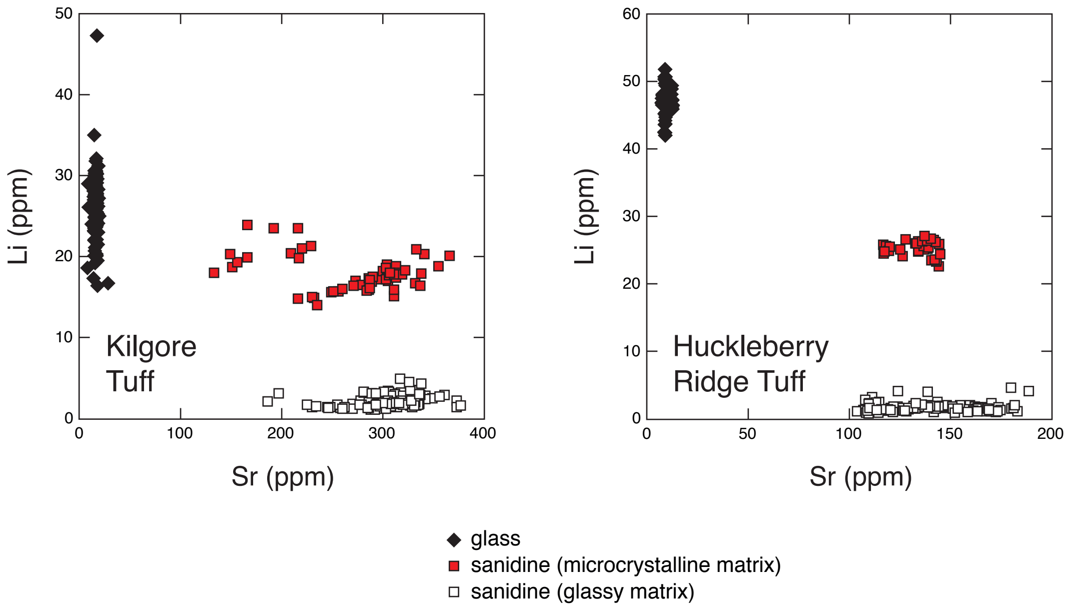

Supplementary Figure 1. Li abundances in sanidine phenocrysts and glass from the Kilgore and Huckleberry Ridge tuffs.

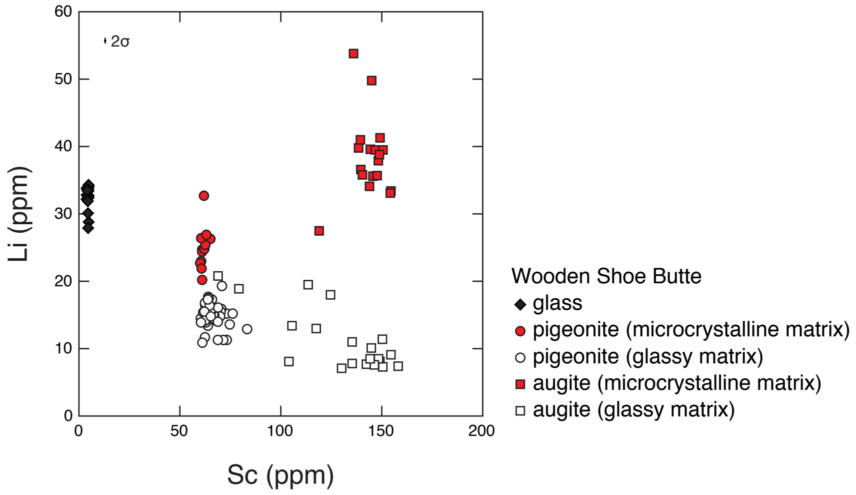

Supplementary Figure 2. Li abundances in clinopyroxene (augite, pigeonite) phenocrysts and glass from the Wooden Shoe Butte Member.

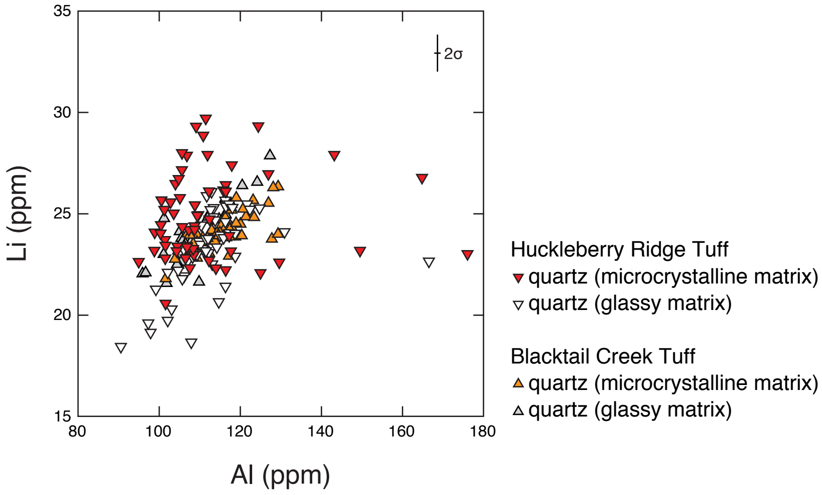

Supplementary Figure 3. Li abundances in quartz from the Huckleberry Ridge and Blacktail Creek tuffs.

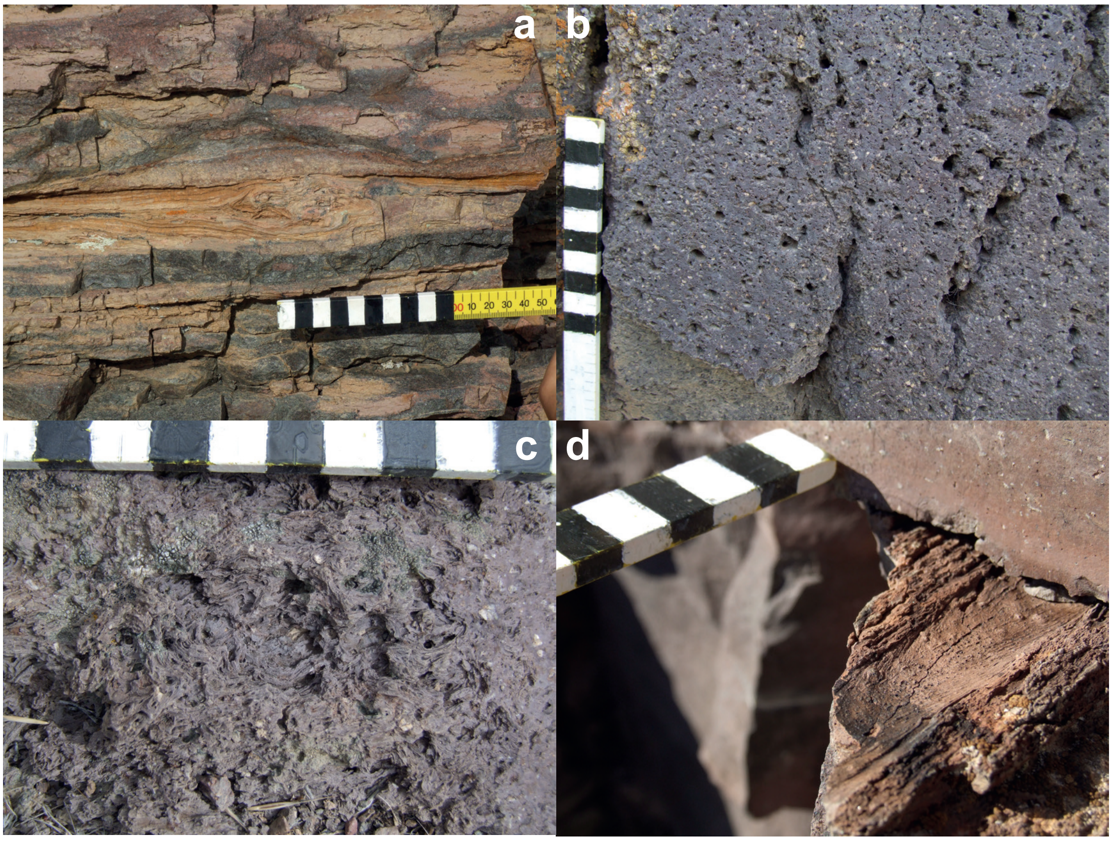

Supplementary Figure 4. Field evidence for post-emplacement degassing of Snake River Plain ignimbrites. **a**, large vesicle in the House Creek ignimbrite, **b–c**, lineated vesicles in the Wooden Shoe Butte Member, **d**, vesicle saddle in Cougar Point Tuff XI.
