# Supplementary material for: Post-eruptive mobility of lithium in volcanic rocks
Source: Nat Commun. 2018 Aug 13;9:3228. doi: 10.1038/s41467-018-05688-2 (PMC6089988; doi:10.1038/s41467-018-05688-2)
Supplement: Supplementary file 2 — Description of Additional Supplementary Files [file 41467_2018_5688_MOESM2_ESM.pdf]

Description of Additional Supplementary Files

File Name: Supplementary Data 1
Description: The file contains all trace elemental and isotopic data that are used in this work and additionally provides results from secondary standards and sample location information.
